# Supplementary material for: Recent decrease of the impact of tropical temperature on the carbon cycle linked to increased precipitation
Source: Nat Commun. 2023 Feb 21;14:965. doi: 10.1038/s41467-023-36727-2 (PMC9944254; doi:10.1038/s41467-023-36727-2)
Supplement: Supplementary file 1 — Supplementary Information [file 41467_2023_36727_MOESM1_ESM.pdf]

Supplementary Information

Recent decrease of the impact of tropical temperature on the carbon cycle linked to increased precipitation

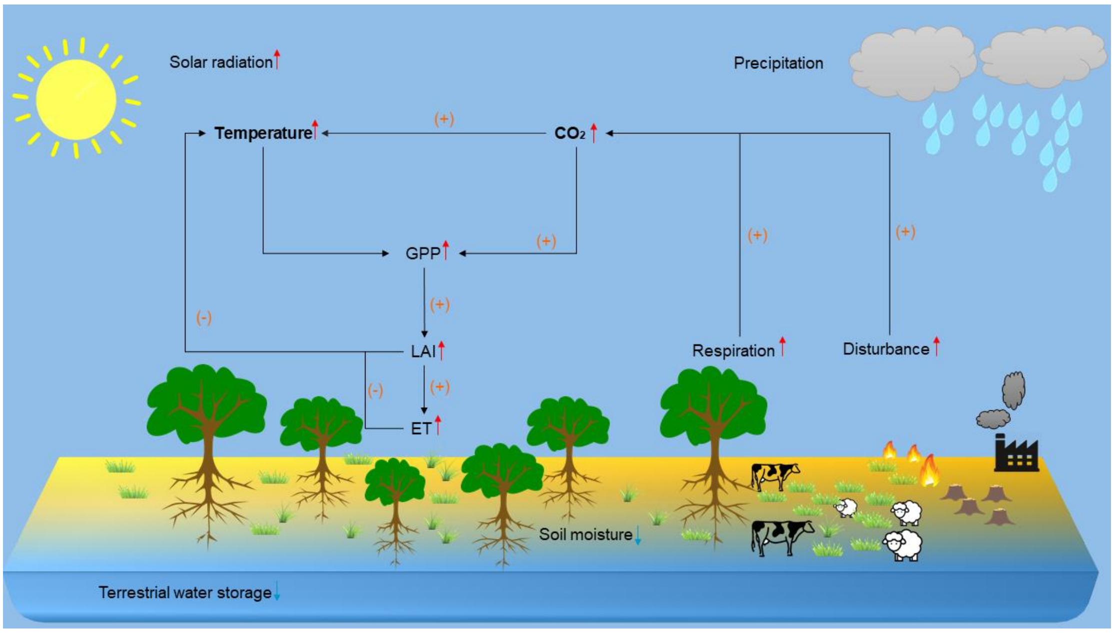

Supplementary Fig. 1 | Interactions of the climate-carbon cycle. + and – indicate positive and negative effects, respectively. LAI: leaf area index; GPP: gross primary productivity; ET: evapotranspiration.

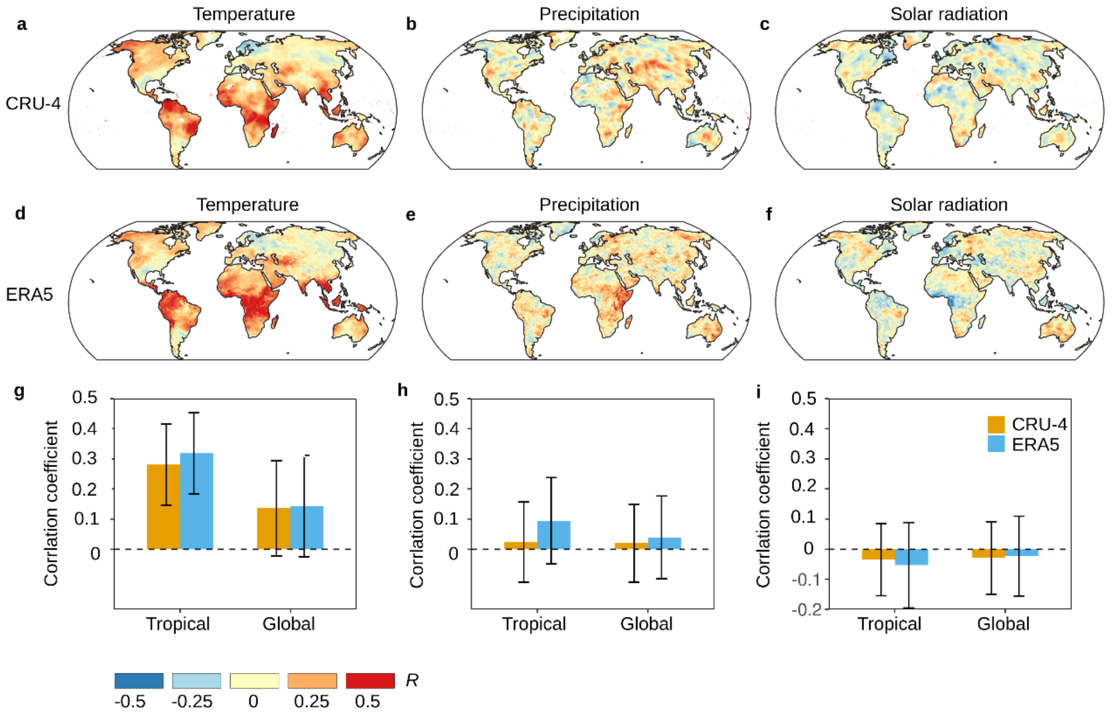

Supplementary Fig. 2 | Spatial patterns of correlations between annual CO_2 growth rate and anomalies of climatic variables for 1960–2020 based on CRU and ERA5 data. a, d, g, CGR-temperature, **b, e, f,** CGR-precipitation, **c, f, g,** CGR-solar radiation (cloud cover was used to indicate solar radiation using CRU data). Partial correlations were used to isolate covarying effects. The error bars in **g-i** represent 1 SD. *R* is the Spearman correlation.

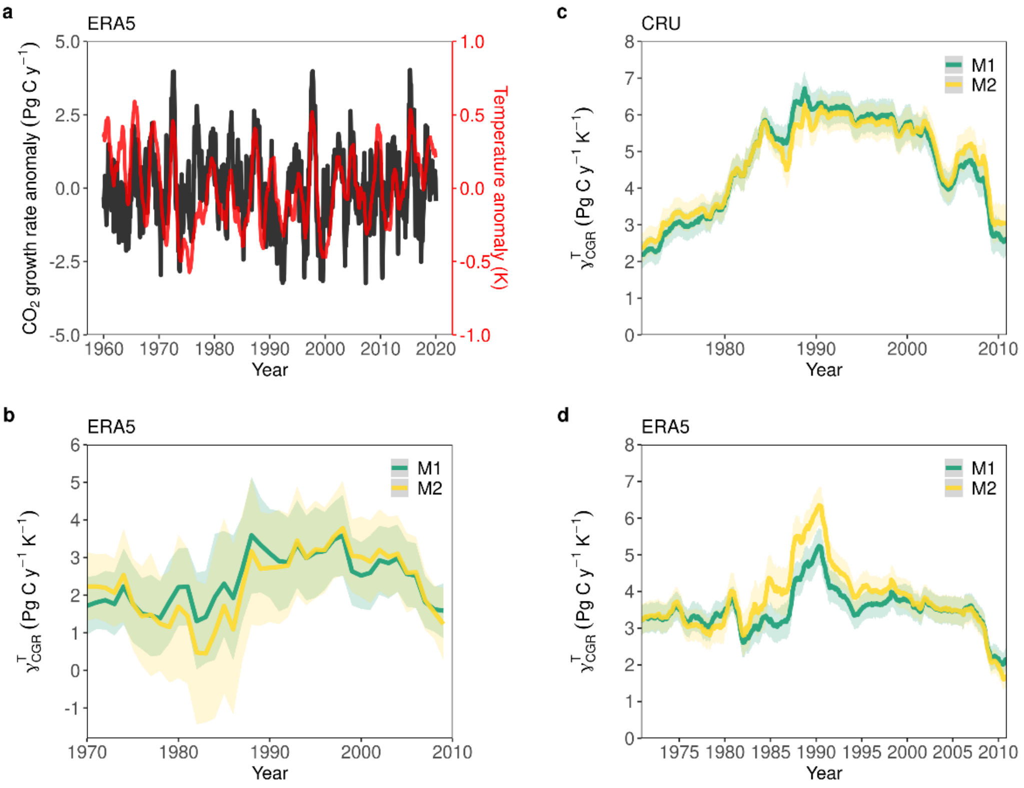

Supplementary Fig. 3 | Changes in detrended anomalies in the CO_2 growth rate and tropical temperature. a and **b,** Same as Fig. 1 in the main manuscript, but with ERA5 climatic data instead of CRU-based climatic data. **c** and **d,** Changes in γ^{T}_{CGR} using a 12-month moving window, based on CRU and ERA5 climatic data, respectively. γ^{T}_{CGR} was calculated with a moving window of 20 y.

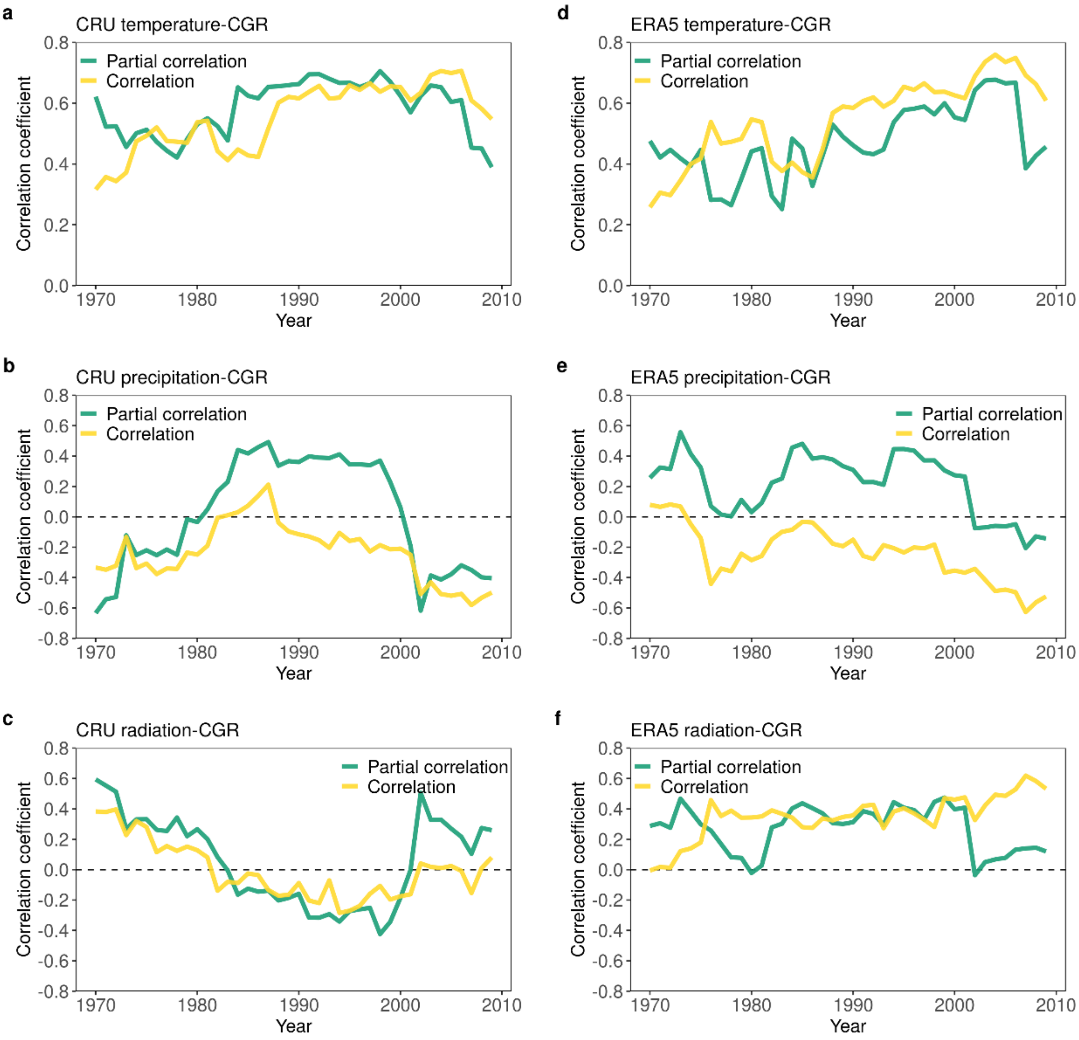

Supplementary Fig. 4 | Correlations between annual CGR and climatic variables. a and **d,** CGR-temperature**, b** and **e,** CGR-precipitation, **c** and **f,** CGR-solar radiation based on CRU and ERA5 climatic data, respectively. Partial correlations were used to isolate the covarying effects between variables.

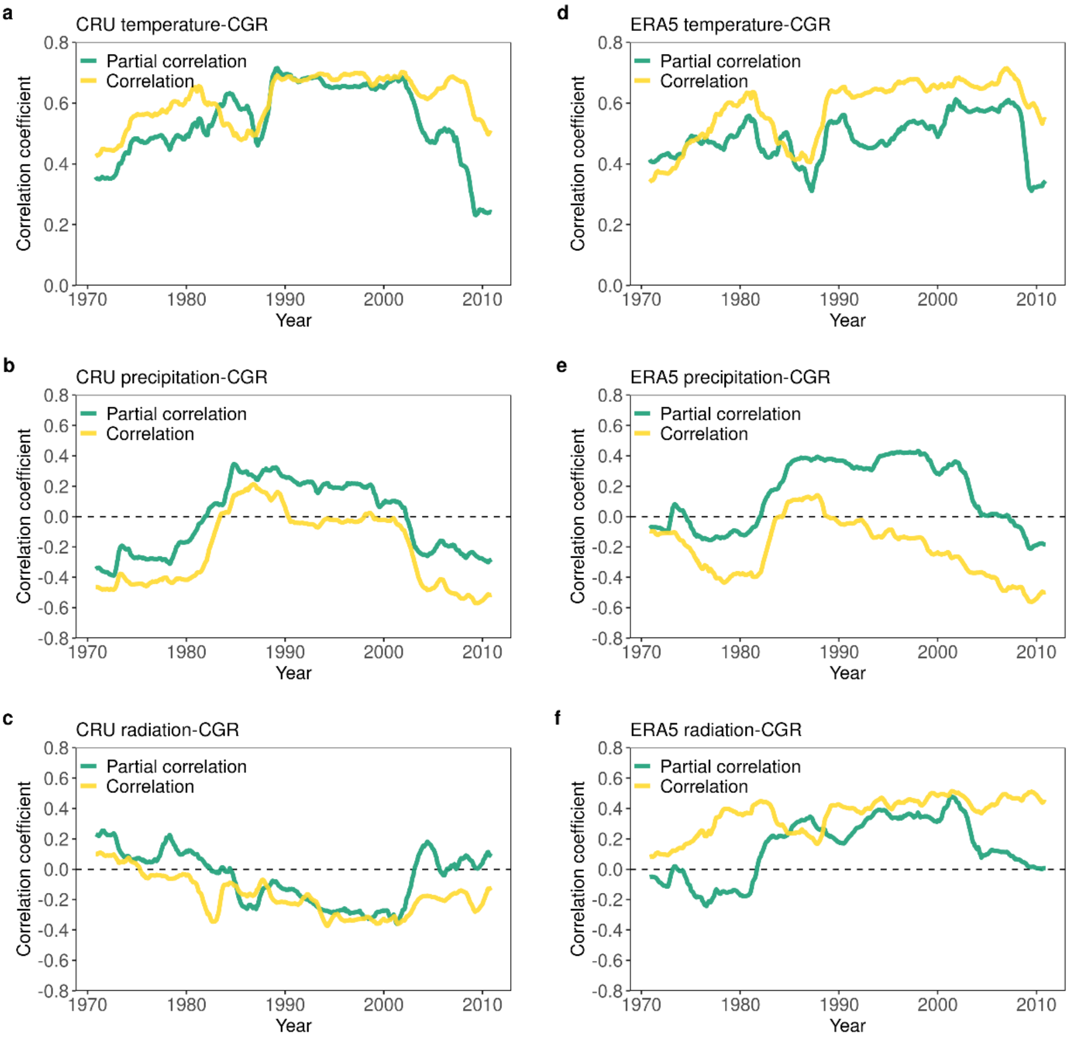

Supplementary Fig. 5 | Correlations between high frequency observations of CGR and climatic variables. a and **d,** CGR-temperature**, b** and **e,** CGR-precipitation, **c** and **f,** CGR-solar radiation based on CRU and ERA5 climatic data, respectively. We calculated CGR and climatic variables at a high temporal frequency using a 12-month moving window (n = 721) and partial correlations were implemented to isolate the covarying effects between variables.

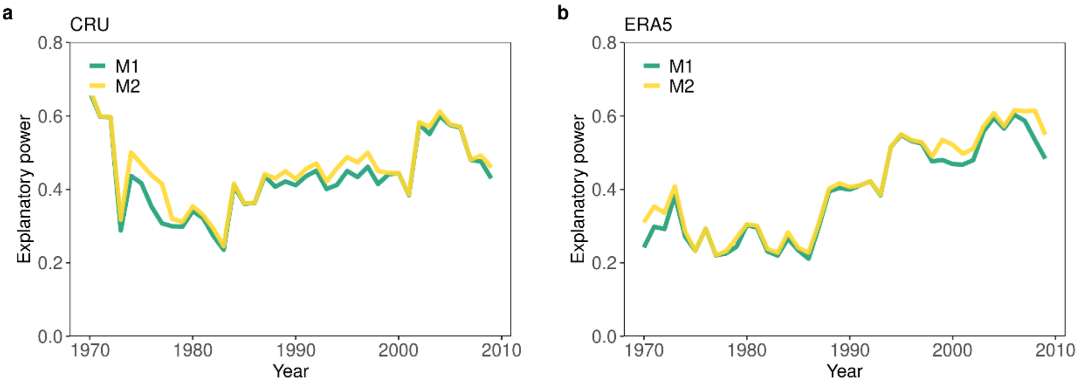

Supplementary Fig. 6 | Explanatory power of climatic variables on CGR. a and **b,** Explanatory power (R^2) of temperature, precipitation and solar radiation on CGR based on CRU and ERA5 climatic data, respectively. The explanatory power was calculated based on multiple regressions of Eq.1-2 with a moving window of 20 y. Temperature, precipitation and solar radiation were used as explanatory variables, and CGR was used as the response variable.

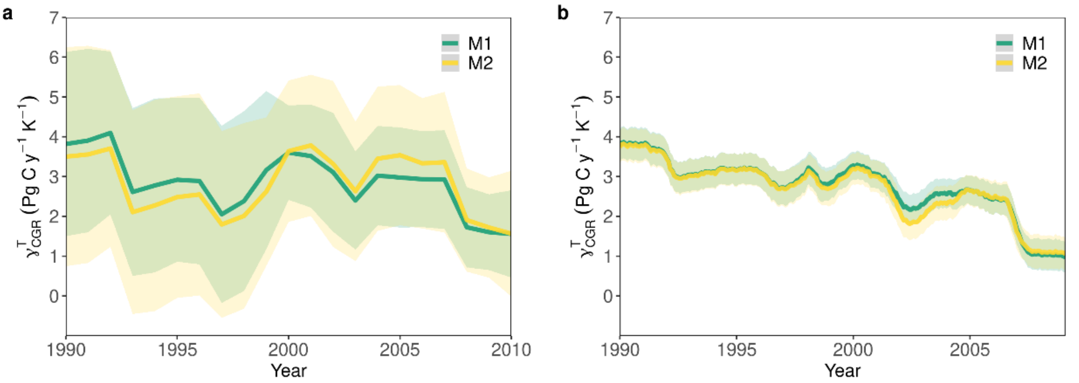

Supplementary Fig. 7 | Changes in the sensitivity of CO_2 growth rate to tropical temperature. a, γ^{T}_{CGR} were calculated with a moving window of 20 years using annual CGR and climatic variables. **b,** same to **a,** but the CO_2 and climatic variables were calculated from a moving window of 12-month moving window. CO_2 data is from the South Pole station and the tropical temperature was derived from the CRU data set for 1981–2020. The shaded areas denote 1 SD of the sensitivity derived from a 20-y moving window in 500 bootstrap estimates. The years on the x-axes indicate the central year of the moving window used to derive γ^{T}_{CGR}.

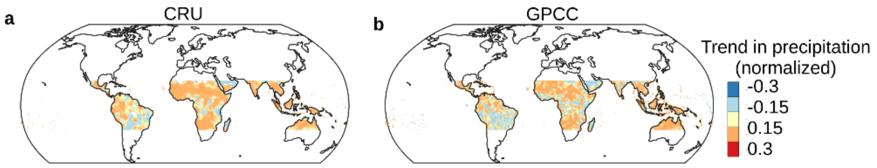

Supplementary Fig. 8 | Trends in bi-decadal precipitation. Trends in bi-decadal mean precipitation based on **a,** CRU (1980–2020) and **b,** GPCC (1980–2019). The bi-decadal precipitation was normalized (z-score) before calculating trends.

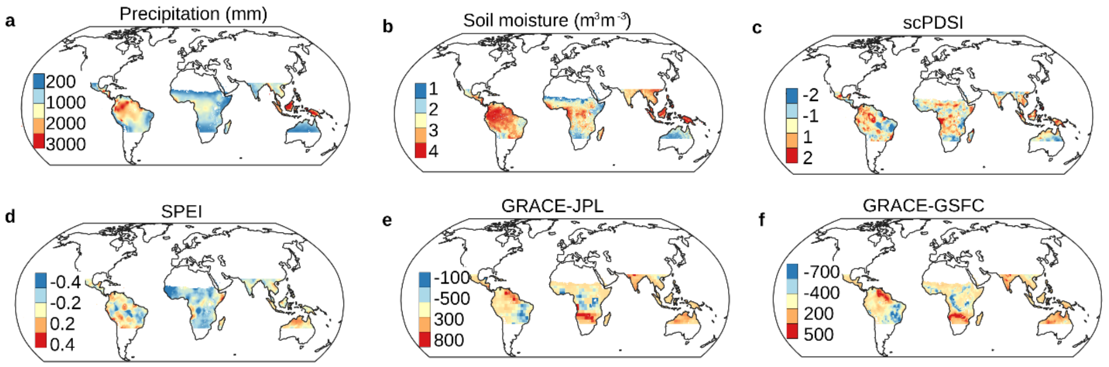

Supplementary Fig. 9 | Spatial patterns of annual mean moisture conditions. a-f, Annual mean precipitation, soil moisture, scPDSI (1980–2020), SPEI and GRACE terrestrial water storage (1980–2018).

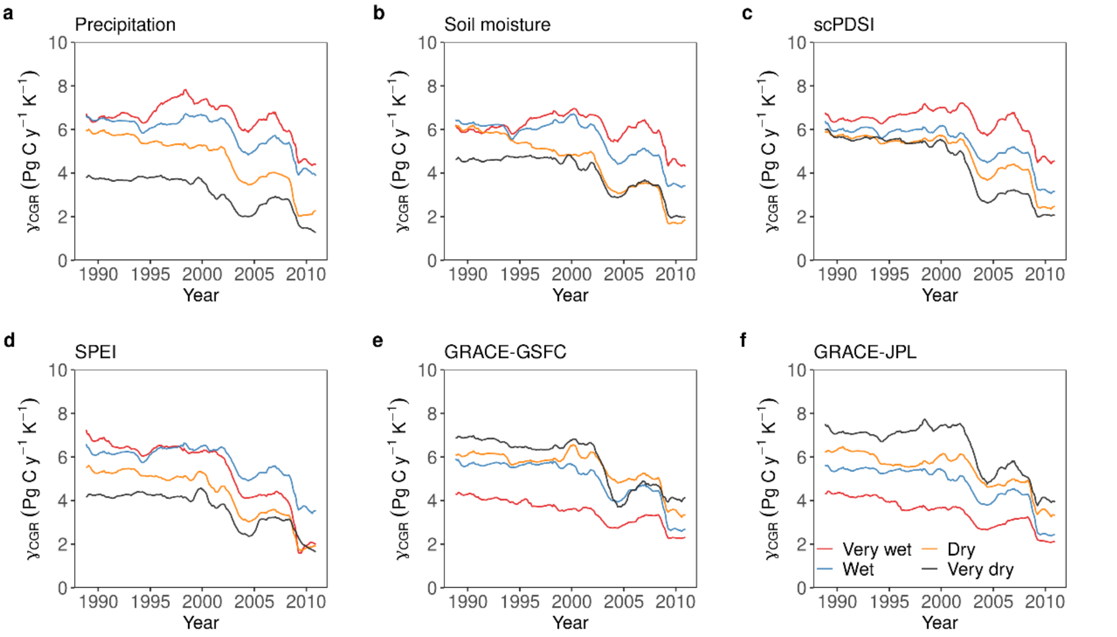

Supplementary Fig. 10 | Changes in γ_{CGR}^{T} under various water gradients for 1978–2020. γ_{CGR}^{T} was calculated based on four bins of detected variables in a 20-y moving window. Very wet ($\sigma \geq 1$), wet ($0 \leq \sigma < 1$), dry ($-1 \leq \sigma < 0$) and very dry ($\sigma < -1$).

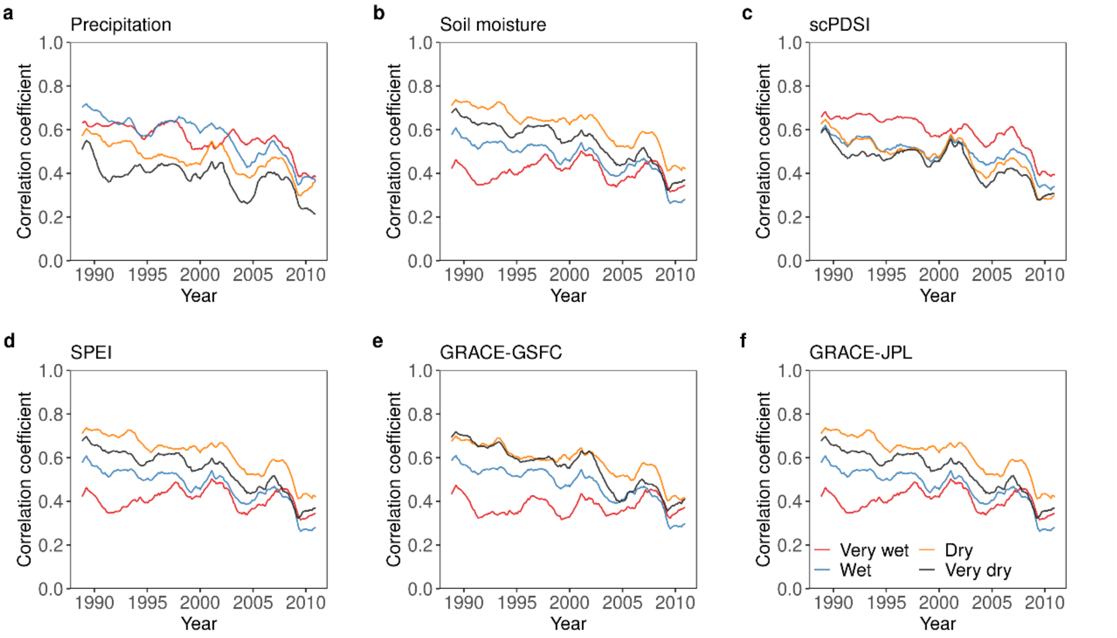

Supplementary Fig. 11 | Changes in the partial correlation between CGR and temperature after controlling for precipitation and solar radiation under various water gradients for 1978–2020. Partial correlations were implemented to isolate the covarying effects and calculated based on four bins of detected variables in a 20-y moving window. Very wet ($\sigma \geq 1$), wet ($0 \leq \sigma < 1$), dry ($-1 \leq \sigma < 0$) and very dry ($\sigma < -1$).

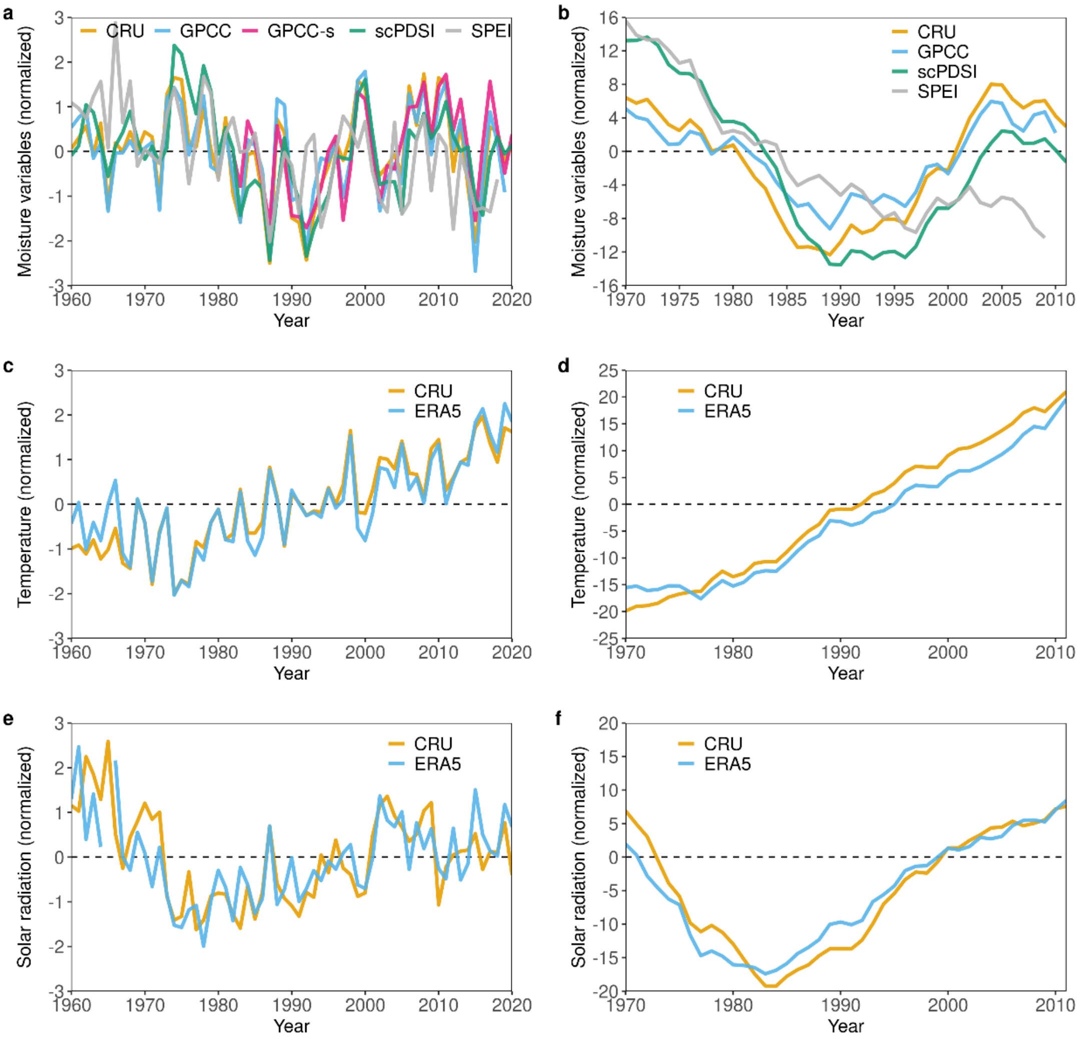

Supplementary Fig. 12 | Changes in water and climatic conditions for 1980–2020. a and **b,** Changes in normalized annual and bi-decadal precipitation, scPDSI and SPEI. **c** and **d** Changes in normalized annual and bi-decadal temperature. **e** and **f,** Changes in normalized annual and bi-decadal solar radiation. Cloud cover from the CRU data set was used to indicate solar radiation with opposite signs.

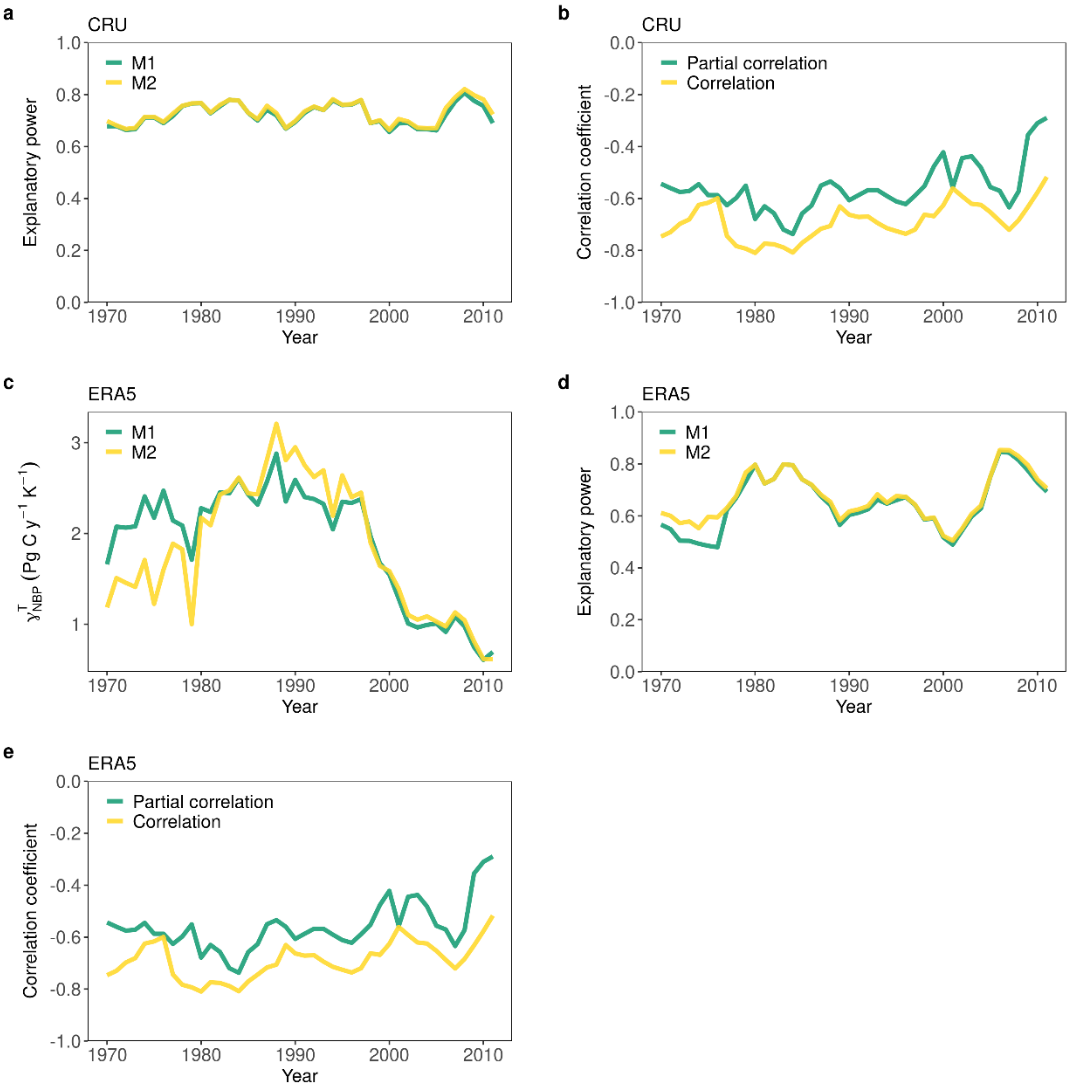

Supplementary Fig. 13 | Changes in relationship between annual NBP and temperature for 1960–2020. a, b, Explanatory power of the annual climatic variables on NBP and the correlation between NBP and temperature based on CRU climatic data. The explanatory power was calculated based on multiple regressions of Eq.1-2 (M1 and M2) with a moving window of 20 y. **c,** Changes in the sensitivity of NBP to temperature using ERA5 climatic data. **d** and **e,** Explanatory power of climatic variables on NBP and the partial correlation between NBP and temperature based on ERA5 climatic data. The explanatory power was calculated based on multiple regression of Eq.1-2 (M1 and M2), with a moving window of 20 y. Temperature, precipitation and solar radiation are used as explanatory variables, and NBP was used as the response variable. Partial correlations were implemented to isolate the covarying effects and were calculated at a 20-y moving window for 1960–2020.

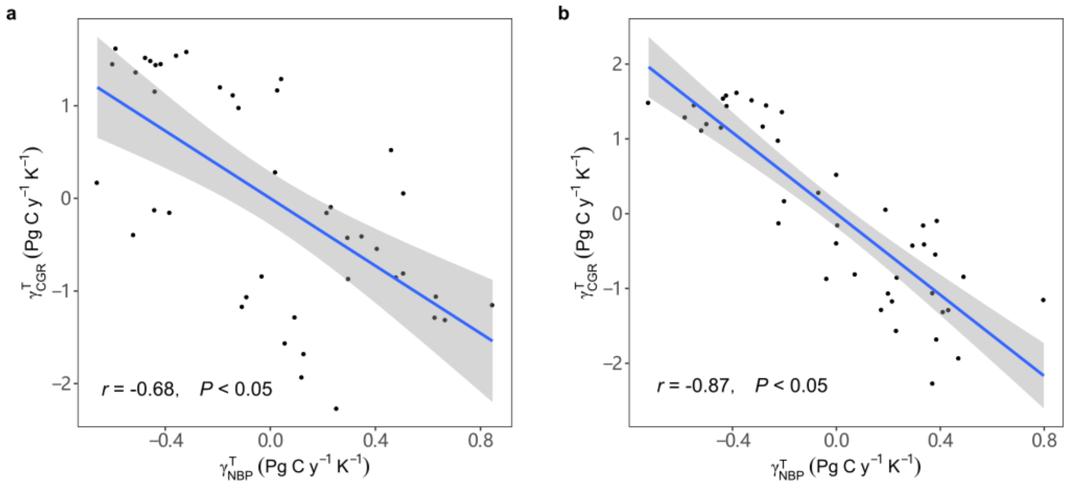

Supplementary Fig. 14 | Relationships between γ^{T}_{CGR} and γ^{T}_{NBP} for 1960–2020. The CGR/NBP sensitivities were computed with Eq.1 under a moving window of 20 y using **a,** CRU and **b,** ERA5 annual climatic data. The sensitivities were detrended, and the shading represents the 95% confidence interval of the fitting.

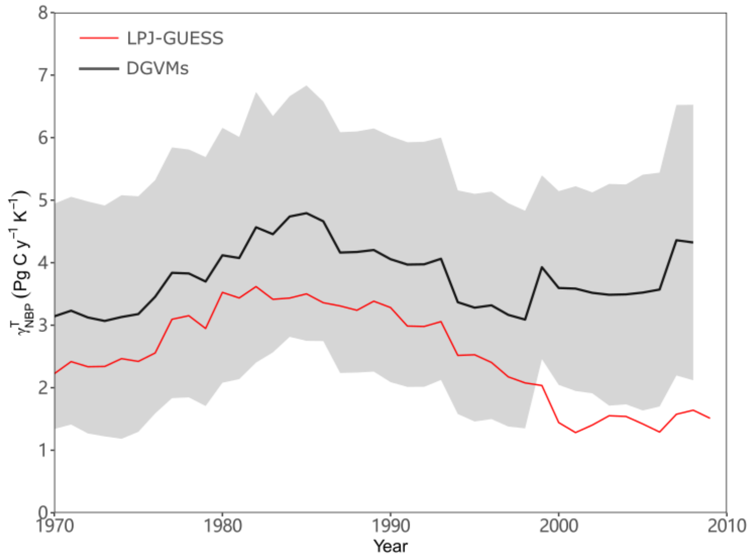

Supplementary Fig. 15 | Changes in the average sensitivities of NBP to tropical temperature during 1960–2020. The NBP sensitivities were computed with CRU annual climatic data. DGVMs from TRENDY version 9 included models of CLASSIC, CLM5.0, DLEM, IBIS, ISBA-CTRIP, JSBACH, JULE-ES, LPJ-GUESS, LPX-Bern, OCN, ORCHIDEE-CNP, ORCHIDEEv3, SDGVM, VISIT, YIBs. The outputs under scenario three (1960–2019) for each model were used in this study. The grey shading represents the mean ± 1 s.d. of NBP sensitivity based on 15 models.

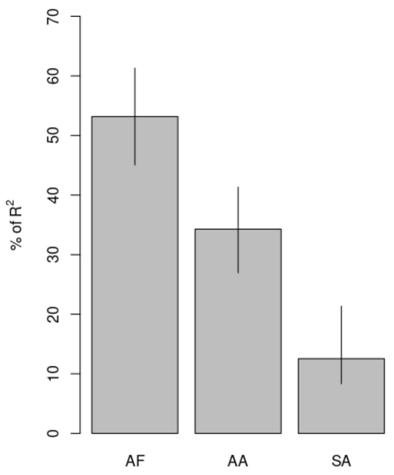

Supplementary Fig. 16 | Contribution to changes in γ^{T}_{NBP} from different tropical regions. Relative importance of the tropical regions of different continents (Africa (AF), Asia-Australia (AA) and South America (SA)) to the changes in γ^{T}_{NBP} for the entire tropical region. Results are based on a multiple linear regression model with γ^{T}_{NBP} of the entire tropical region used as a response variable and γ^{T}_{NBP} of the different regions used as explanatory variables. The explanatory power is 95% and the relative importance is assessed using the "lmg" approach, which is based on sequential R^2 but accounts for the dependence on ordering of explanatory variables. Error bars denoted 1 SD of the explanatory power in 500 bootstrap estimates.

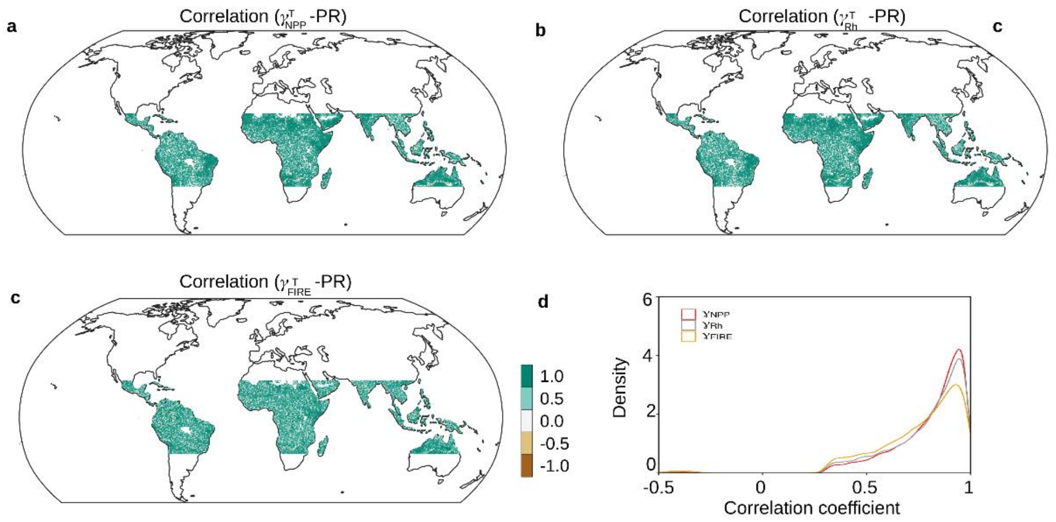

Supplementary Fig. 17 | Carbon flux component sensitivity to tropical temperature in relation to precipitation as simulated in LPJ-GUESS. a-c, Spatial patterns of correlation coefficients of γ^{T}_{NPP}, γ^{T}_{Rh} and γ^{T}_{FIRE} under scenarios of SCE1 (all drivers of NBP vary with time) and SCE1 - SCE2 (precipitation drives variations in NBP). Pixels with significant ($P < 0.05$) correlation are shown. **d,** Density plot of the distributions of correlation coefficients.

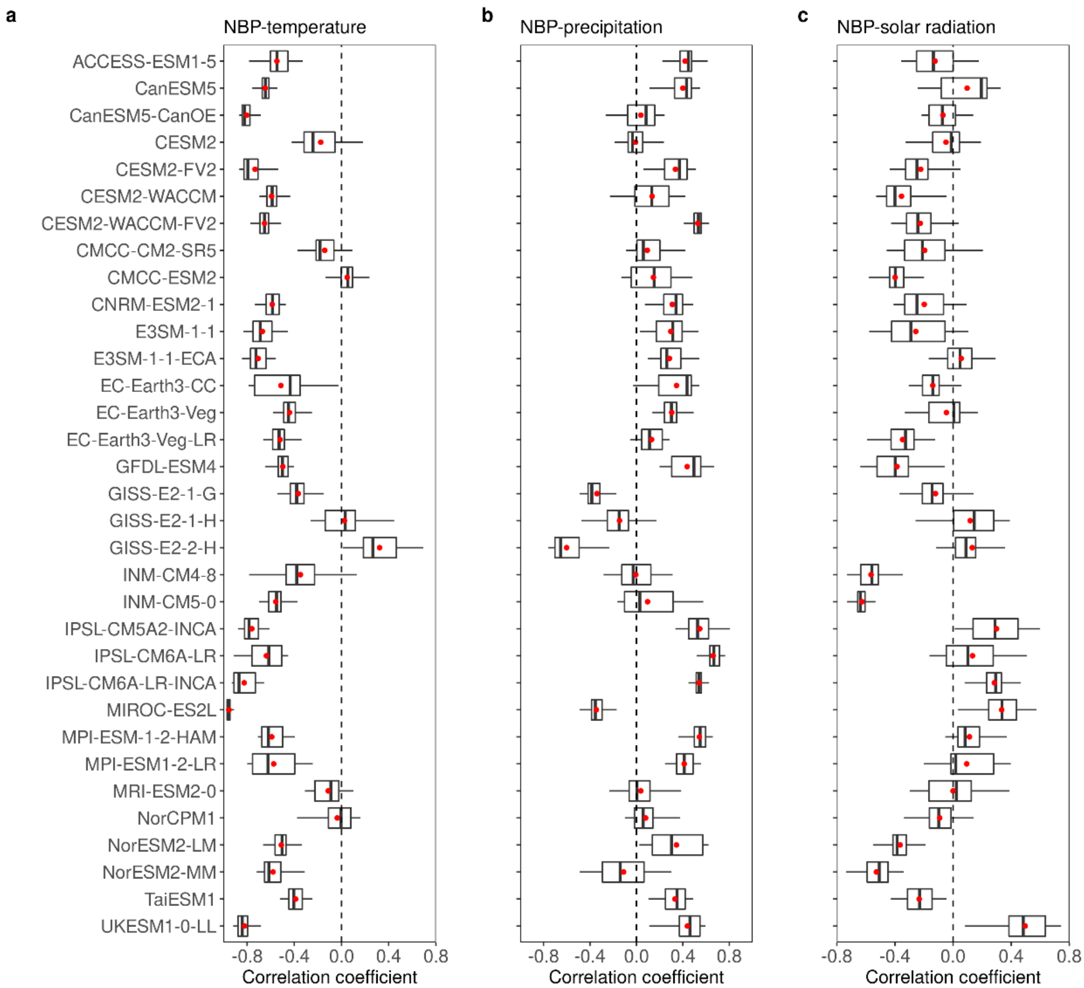

Supplementary Fig. 18 | Correlations between NBP and tropical temperature simulated in CMIP6. a-c, Partial correlations between annual NBP and temperature, precipitation and solar radiation. Partial correlations were implemented to isolate the covarying effects and were calculated using a 20-y moving window for 1960–2014. The vertical lines in the box plots represent, from right to left, the 95th, 75th, 50th, 25th and 5th percentiles, and the red dots represent the average correlation coefficients.

Supplementary Table 1 | Partial correlations between CGR sensitivity, bi-decadal CGR, temperature (TMP) and precipitation (PR). The sensitivities were calculated using a multiple regression approach referring to Eq.1 in a moving window of 20 y. Bi-decadal CGR, TM and PR were averaged per 20-y moving window.

	**CGR sensitivity**	**CGR**	**TMP**	**PR**
**CGR sensitivity**	1	-0.45	-0.07	**-0.88**
**CGR**		1	-0.31	-0.50
**TMP**			1	0.01
**PR**				1

Supplementary Table 2 | Cochrane-Ocrutt test with the time series accounting for serial autocorrelations. Durbin-Watson indicators (DWI) ranges between 0 and 4. A value of 2 indicates there is no autocorrelation detected. Values from 0 to less than 2 denotes positive autocorrelation and values from 2 to 4 denotes negative autocorrelation. The significance level of $P < 0.001$ was set.

	**CGR**	**PR**	**TMP**	**DWI**
**Estimate coefficients**	-1.95	-0.08	-9.48	0.66 (original)
***P* value**	0.04	$\mathbf{7.0 \times 10^{-8}}$	0.13	1.82 (transformed)

Supplementary Table 3 | Schemes of simulations using the LPJ-GUESS model. SCE, scenario; TMP, air temperature; PR, precipitation; SR, solar radiation; ND: nitrogen deposition, LC: land cover change. T-V, time-varying; CONS, constant.

	**TMP**	**PR**	**SR**	**CO_2**	**LC**	**ND**
SCE1	T-V	T-V	T-V	T-V	T-V	T-V
SCE2	T-V	CONS	T-V	T-V	T-V	T-V
SCE3	T-V	T-V	CONS	T-V	T-V	T-V
SCE4	T-V	T-V	T-V	CONS	T-V	T-V
SCE5	T-V	T-V	T-V	T-V	CONS	T-V
SCE6	T-V	T-V	T-V	T-V	T-V	CONS

Supplementary Table 4 | CMIP6 models used in this study. In total, 33 ESMs with the modelled variables of net biome productivity, air temperature, precipitation, solar radiation are available and used in this study. Solar radiation was calculated as the difference between surface down- and upwelling shortwave radiation simulated in CMIP6.

	**Model**	**Nominal grid resolution**	**Land component**	**Labels**
1	ACCESS-ESM1-5	1.875°×1.25°	CABLE2.4	B
2	CanESM5-CanOE	2.81°×2.81°	CLASS3.6&CTEM1.2	D
3	CanESM5	2.81°×2.81°	CLASS3.6&CTEM1.2	C
4	CESM2-FV2	0.9°×1.25°	CLM5	E
5	CESM2	0.9°×1.25°	CLM5	
6	CESM2-WACCM	0.9°×1.25°	CLM5	F
7	CESM2-WACCM-FV2	0.9°×1.25°	CLM5	G
8	CMCC-CM2-SR5	0.9°×1.25°	CLM4.5	
9	CMCC-ESM2	0.9°×1.25°	CLM4.5	
10	GISS-E2-1-G	2°×2.5°	GISS-LSM	
11	GISS-E2-1-H	2°×2.5°	GISS-LSM	
12	GISS-E2-2-H	2°×2.5°	GISS-LSM	
13	MIROC-ES2L	2.81°×2.81°	MATSIRO6.0	T
14	MPI-ESM1-2-LR	1.52°×0.82°	JSBACH3.20	U
15	MPI-ESM-1-2-HAM	1.52°×0.82°	JSBACH3.20	V
16	MRI-ESM2-0	1.41°×1.41°	HAL 1.0 &MRI-LCCM2	W
17	NorESM2-LM	1.88°×3.25°	CLM5	Y
18	NorESM2-MM	1.88°×3.25°	CLM5	Z
19	TaiESM1	0.9°×1.25°	CLM4.0	a
20	UKESM1-0-LL	1.88°×1.25°	JULES-HadGEM3-GL7.1	b
21	NorCPM1	1.9°×2.5°	CLM4.0	X
22	CNRM-ESM2-1	1°×1°	ISBA-CTRIP	H
23	GFDL-ESM4	2°×2.5°	GFDL-LM4.0.1	N
24	IPSL-CM5A2-INCA	2.5°×1.25°	ORCHIDEE	Q
25	IPSL-CM6A-LR	2.5°×1.25°	ORCHIDEE	R
26	IPSL-CM6A-LR-INCA	2.5°×1.25°	ORCHIDEE	S
27	E3SM-1-1	1°×1°	ELM1.0&E3SM	I
28	E3SM-1-1-ECA	1°×1°	ELM1.0E3SM	J
29	EC-Earth3-CC	0.7°×0.7°	H-TESSEL&LPJ-GUESS	K
30	EC-Earth3-Veg	0.7°×0.7°	H-TESSEL&LPJ-GUESS	L
31	EC-Earth3-Veg-LR	0.7°×0.7°	H-TESSEL&LPJ-GUESS	M
32	INM-CM4-8	2°×1.5°	INM-LND1	O
33	INM-CM5-0	2°×1.5°	INM-LND1	P
